# Supplementary material for: Simultaneous separation of naproxen and 6-O-desmethylnaproxen metabolite in saliva samples by liquid chromatography–tandem mass spectrometry: Pharmacokinetic study of naproxen alone and associated with esomeprazole
Source: PLoS One. 2020 Aug 11;15(8):e0236297. doi: 10.1371/journal.pone.0236297 (PMC7418953; doi:10.1371/journal.pone.0236297)
Supplement: Attachment — Submitted filename: Answer to reviewers 2.docx [file pone.0236297.s001.docx]

**Answer to reviewers**

Reviewer #1: This manuscript does not appear complete, as there are no analyses, results, or discussion sections. It appears to only be a partly-developed protocol as there is no mention of any planned statistical analyses for the data collected from 17 time points. This needs to be amended, and results should be shown if it is to be a true P/K study.

Additionally, it is mentioned that time point 0 for each drug were analyzed to ensure no interference, but there is no mention of how this analysis was performed.

**Answer: Thank you for your observation. The statistical analyses, which we intend to use, were added in the method section. Our article type is Registered Report Protocol, therefore, the results will be presented in the second manuscript as well as the discussion. The time point 0 refers to the collection of saliva from the volunteers before taking the medications. In fact, this collection is important to ensure that the pre-determined methods of detecting the drugs under study do not detect other molecules in the volunteers' saliva.**

Reviewer #2: The submission was not complete as only the abstract, introduction and methods were provided. Despite this, the following major issues were identified which must be addressed in the complete version:
• The method is intended for a clinical application among patients. Method validation is a must- it is not optional when dealing with patient samples. There was no mention of method validation. The authors must completely validate the method as per regulatory guidelines, the FDA, EMA or ICH.

**Answer: Thank you for your observation. Our article type is Registered Report Protocol, therefore, the results will be presented in the second manuscript as well as the discussion (if this manuscript is able to be accepted). We will we will execute the validations in all analyzes carried out in accordance with the FDA Please find the description of the experiments for validation in the methods section.**

• Though the authors indicate that this is the first time a method is reported for Saliva, did they attempt the conditions of the reported methods measuring the same compounds. Is there any similarities or improvements form an analytical stand point?

**Answer: Thank you for your question. Yes, we did a research where we compared the detection and quantification of Piroxicam in saliva and plasma. These results confirmed that in saliva it is possible to perform pharmacokinetic tests as in plasma (Calvo et al. J Pharm Biomed Anal. 2016 Feb 20;120:212-20. doi: 10.1016/j.jpba.2015.12.042).**

• Patient sample preparation [containing the drug] was not adequately described.

**Answer: Thank you for your observation. We adequately described patient sample preparation. Please find this new information in method section.**

• PK studies are usually done in plasma- what information we can get from a Saliva for a medication intended for pain. Clarification and justification are needed to justify publication.

**Answer: Thank you for your question. The idea of the study is to share the experience that it is possible to perform pharmacokinetic tests, using saliva, of any drug, including anti-inflammatory drugs. Pharmacokinetic studies are important for planning treatment according to the speed of metabolism of each person. This information was added in the introduction.**

• What is the structures of the drugs and their monitored product ions? MSMS data should be shown along with the proposed structures. Why these specific product ions were chosen?

**Answer: Thank you for your observation. Our mass spectrometer, based on the reported drug mass, automatically optimizes all parameters including product ions, which can be seen in Table 1.**

• Did the authors monitor the ratios of the quantifier/qualifier ions? A must when doing quantification with LC-MS/MS to ensure peak purity.

**Answer: Thank you for your observation. These information is in the new table 2.**

**Table 2: Quantifier and qualifier product ion to be used in analysis.**

| **O-Desmethylnaproxen** | | | | |
| --- | --- | --- | --- | --- |
| **Type** | **m/z** | **Inten** | **Act %** | **Idenf. Rang %** |
| T | 214.900>171.250^a^ | 199246 | 100.00 | 100% |
| Ref 1 | 214.900>169.250^b^ | 49605 | 24.90 | - |
| Ref 2 | 214.900>143.050 | 3283 | 1.65 | - |
|  |  |  |  |  |
| **Naproxen** | | | | |
| **Type** | **m/z** | **Inten** | **Act %** | **Idenf. Rang %** |
| T | 228.900>170.30^a^ | 513749 | 100.00 | 100% |
| Ref 1 | 228.900>169.250^b^ | 336855 | 65.57 | - |
| Ref 2 | 228.900>185.200^b^ | 288128 | 56.08 | - |
|  |  |  |  |  |
| **Piroxicam** | | | | |
| **Type** | **m/z** | **Inten** | **Act %** | **Idenf. Rang %** |
| T | 331.900>95.100^a^ | 33688 | 100.00 | 100% |
| Ref 1 | 331.900>121.050 | 15274 | 45.37 | - |
| Ref 2 | 331.900>78.100^b^ | 23870 | 70.90 | - |

aQuantifier - the most intense peak

bQualifier - a second peak (SRM)t have used as a qualifier for the same analysis

• In fact, it was not clear which product ion was the quantifier and which one is the qualifier

**Answer: Thank you for your observation. Please check the answer above.**

• The authors mention molecular ions. I think the authors mean protonated ions as molecular ions are the radical cations and it is not commonly seen in ESI.

**Answer: Thank you for your observation. Sorry for the mistake. In fact we refer to the protonated ions.**

• Why a structural analogue was used for internal standard instead of isotopically labelled IS.

**Answer: Thank you for your question. Previous studies by our group have shown that Piroxicam could be used successfully as an internal standard. However, we have the conscience and the intention to start using the isotopically labeled IS. During the validation process, we will guarantee that there will be no interference from the chosen IS.**

**Answer to editors**

1. We suggest you thoroughly copyedit your manuscript for language usage, spelling, and grammar. If you do not know anyone who can help you do this, you may wish to consider employing a professional scientific editing service. 

Whilst you may use any professional scientific editing service of your choice, PLOS has partnered with both American Journal Experts (AJE) and Editage to provide discounted services to PLOS authors. Both organizations have experience helping authors meet PLOS guidelines and can provide language editing, translation, manuscript formatting, and figure formatting to ensure your manuscript meets our submission guidelines. To take advantage of our partnership with AJE, visit the AJE website (<http://learn.aje.com/plos/>) for a 15% discount off AJE services. To take advantage of our partnership with Editage, visit the Editage website ([www.editage.com](http://www.editage.com)) and enter referral code PLOSEDIT for a 15% discount off Editage services.  If the PLOS editorial team finds any language issues in text that either AJE or Editage has edited, the service provider will re-edit the text for free.

Upon resubmission, please provide the following:
●       The name of the colleague or the details of the professional service that edited your manuscript.

**Answer: Thank you for your suggestion. We contracted the correction and editing service from Editage (https://www.editage.com.br). Follows the certificate issued by the company attached in cover letter.**

●       A copy of your manuscript showing your changes by either highlighting them or using track changes (uploaded as a *supporting information* file)

**Answer: Thank you for your suggestion. A track changed manuscript was uploaded as supporting information.**

●       A clean copy of the edited manuscript (uploaded as the new *manuscript* file)

**Answer: Thank you for your suggestion. A clean copy of the edited manuscript was uploaded as manuscript file.**

2. Thank you for stating the following in the Acknowledgments Section of your manuscript:
We are grateful for São Paulo Research Foundation’s (FAPESP) financial support. Grant #2017/12725-0.
We note that you have provided funding information that is not currently declared in your Funding Statement. However, funding information should not appear in the Acknowledgments section or other areas of your manuscript. We will only publish funding information present in the Funding Statement section of the online submission form.
Please remove any funding-related text from the manuscript and let us know how you would like to update your Funding Statement. Currently, your Funding Statement reads as follows:
"The funders had and will not have a role in study design, data collection and analysis, decision to publish, or preparation of the manuscript."

**Answer: Thank you for your observation. We removed funding information from the acknowledgements. The currently Funding Statement is correct. We would like to thank FAPESP (Grant #2017/12725-0) for the financial support for this research in an appropriate field.**

3. To comply with PLOS ONE submission guidelines, in your Methods section, please provide additional information regarding your statistical analyses that will be conducted in your study. For more information on PLOS ONE's expectations for statistical reporting, please see <https://journals.plos.org/plosone/s/submission-guidelines.#loc-statistical-reporting>

**Answer: Thank you for your suggestion. Please find the following statement in method section. After analyzing the PK and pharmacodynamic results using the Phoenix WinNonlin software (version 8.1), the data will be organized descriptively and a paired t-test will be performed to compare naproxen absorption after volunteers are administered, at two different times, naproxen alone or naproxen in combination with esomeprazole.**

4. We note that you have stated that you will provide repository information for your data at acceptance. Should your manuscript be accepted for publication, we will hold it until you provide the relevant accession numbers or DOIs necessary to access your data. If you wish to make changes to your Data Availability statement, please describe these changes in your cover letter and we will update your Data Availability statement to reflect the information you provide.

**Answer: Thank you for your observation. We do not wish to make changes to our data availability statement.**

5. It appears that your ORCiD iD has not been validated in your Editorial Manager account and we are unable to proceed until that step is complete.

**Answer: Thank you for your observation. We validated the ORCiD iD in Editorial Manager.**
